# *Aeromonas dhakensis*: Clinical Isolates with High Carbapenem Resistance

**DOI:** 10.3390/pathogens11080833

**Published:** 2022-07-26

**Authors:** Suat Moi Puah, Wei Ching Khor, Kyaw Thu Aung, Tien Tien Vicky Lau, S. D. Puthucheary, Kek Heng Chua

**Affiliations:** 1 Department of Biomedical Science, Faculty of Medicine, University of Malaya, Kuala Lumpur 50603, Malaysia; suatmoi@um.edu.my (S.M.P.); vickylau92@hotmail.com (T.T.V.L.); puthucheary@gmail.com (S.D.P.); 2 National Centre for Food Science, Singapore Food Agency, 52 Jurong Gateway Road, JEM Office Tower, 14-01, Singapore 608550, Singapore; khor_wei_ching@sfa.gov.sg (W.C.K.); aung_kyaw_thu@sfa.gov.sg (K.T.A.); 3 School of Biological Sciences, Nanyang Technological University, 60 Nanyang Drive, Singapore 637551, Singapore

**Keywords:** *Aeromonas dhakensis*, carbapenem, CLSI, EUCAST, MicroScan

## Abstract

*Aeromonas dhakensis* is ubiquitous in aquatic habitats and can cause life-threatening septicaemia in humans. However, limited data are available on their antimicrobial susceptibility testing (AST) profiles. Hence, we aimed to examine their AST patterns using clinical (*n* = 94) and non-clinical (*n* = 23) isolates with dehydrated MicroScan microdilution. Carbapenem resistant isolates were further screened for genes related to carbapenem resistance using molecular assay. The isolates exhibited resistance to imipenem (76.9%), doripenem (62.4%), meropenem (41.9%), trimethoprim/sulfamethoxazole (11.1%), cefotaxime (8.5%), ceftazidime (6%), cefepime (1.7%) and aztreonam (0.9%), whereas all isolates were susceptible to amikacin. Clinical isolates showed significant association with resistance to doripenem, imipenem and meropenem compared to non-clinical isolates. These *bla*_cphA_ were detected in clinical isolates with resistance phenotypes: doripenem (67.2%, 45/67), imipenem (65.9%, 54/82) and meropenem (65.2%, 30/46). Our findings showed that the MicroScan microdilution method is suitable for the detection of carbapenem resistance in both clinical (48.9–87.2%) and non-clinical (4.3–13.0%) isolates. This study revealed that *A. dhakensis* isolates had relatively high carbapenem resistance, which may lead to potential treatment failure. Continued monitoring of aquatic sources with a larger sample size should be carried out to provide further insights.

## 1. Introduction

*Aeromonas* species are ubiquitous, Gram-negative, facultative anaerobes, which can cause a variety of infections in poikilothermic animals and humans [1]. They can be isolated from virtually all environmental niches where bacterial ecosystems exist (aquatic habitats, fish, foods, domesticated pets, invertebrate species, birds, ticks, insects and natural soils). The number of species in the genus increased rapidly in the age of molecular genetics and consists of 36 species reported to date [2]. The exact incidence of *Aeromonas* infection in humans on a global basis is limited, as many cases are undetected or not reported.

*Aeromonas dhakensis* (previously named *A. hydrophilla* or *A. aquariorum*) is emerging as a clinically important pathogen that can cause severe soft tissue degloving infections arising from occupational and recreational hazards, as well as bloodstream infections in immunocompromised individuals with malignancy and cirrhosis [3]. A higher mortality rate was observed in *A. dhakensis* bacteraemia compared to bacteraemia caused by non-*A.* *dhakensis* species [4]. Researchers reported greater virulence properties in *A. dhakensis* than other species, such as more robust biofilm formation and a lower survival rate in *Caenorhabditis elegans*, and some strains exhibited a higher minimum inhibitory concentration (MIC) for certain antimicrobial agents [4]. Recently, two fatal cases of *A. dhakensis* bacteraemia and necrotising fasciitis in severe dengue patients were reported in Southern Taiwan, and a fatal case of *A. dhakensis* septicaemia in a hepatitis B virus-infected patient after the ingestion of a meal of raw snakehead fish was reported in China [5,6].

*A. dhakensis* has been misidentified as *A. hydrophila* or *A. caviae* by phenotypic methods in the past decade. Our previous studies conducted since 2012 identified *A. dhakensis* as the predominant species (47/94, 50%) among clinical isolates in Malaysia by using a combination gene analysis of *GCAT* and *rpoD* genes, as well as multilocus sequence typing [7,8]. Following these studies, the presence of *A. dhakensis* was reported from aquatic sources in Malaysia, including multipurpose freshwater recreational lakes in Selangor [9], tank water of ornamental fish in Klang Valley [10], food fish in East Malaysia [11] and as clinical isolates of *A. dhakensis* in Singapore [12]. All aforementioned studies shed light on the clinical relevance of *A. dhakensis* and its ability to present/colonise various sources posing public health concerns. Recognising its clinical relevance and capability of causing invasive disease, understanding the antimicrobial resistance (AMR) profile of *A. dhakensis* is important to support the selection of optimal treatment regimens. In this study, we examined the antimicrobial resistance patterns of *A. dhakensis* from clinical and non-clinical sources (ornamental fish tank water, freshwater from a recreational lake and food fish) based on minimum inhibitory concentrations (MICs) using the broth microdilution method.

## 2. Results

### 2.1. AST Patterns

AST results for 18 antimicrobial agents were interpreted according to the CLSI 2015 (Table 1). All isolates were susceptible to amikacin. The top 8 resistance patterns observed in 117 *A. dhakensis* were present in the following proportions: imipenem, 76.9%; doripenem, 62.4%; meropenem, 41.9%; trimethoprim/sulfamethoxazole, 11.1%; cefotaxime, 8.5%; ceftazidime, 6.0%; cefepime, 1.7%; and aztreonam, 0.9% (Table 1). The MIC range for imipenem and meropenem was 4 to >8 µg/mL, and most *A. dhakensis* isolates with >8 µg/mL were observed for imipenem (Malaysia clinical isolates, 72.3% (34/47); Singapore clinical isolates, 90.2% (37/41); non-clinical isolates, 26.1% (6/23)) compared to meropenem. The resistant isolates in our study showed MIC values towards antimicrobial agents as follow: cefotaxime >4 to >32 μg/mL; ceftazidime and aztreonam, >16 μg/mL; cefepime, 16 μg/mL; doripenem, 4 to 4 μg/mL; imipenem and meropenem, 4 to 8 μg/mL; and trimethoprim/sulfamethoxazole, >4/76 μg/mL.

Our study revealed that clinical *A. dhakensis* isolates exhibited > 2.5-fold significantly higher carbapenem resistance than non-clinical isolates, i.e., doripenem, 71.3% vs. 26.1% (*p*-value = 0.0001); imipenem, 87.2% vs. 34.8% (*p*-value = 0.0001); and meropenem, 48.9% vs. 13.0% (*p*-value = 0.0019). Further comparison between the clinical isolates from Malaysia and Singapore (Table 2) showed comparable resistance rates against carbapenems: doripenem, 70.2% and 72.3%; imipenem, 87.2% for both countries; and meropenem, 48.9% in both countries. However, Singapore had a significantly higher proportion of resistance than Malaysia’s clinical isolates to cefotaxime (19.1% vs. 0%) and ceftazidime (14.9% vs. 0%). Six clinical *A. dhakensis* recovered from Singapore (one stent, one bile drain, one fluid, one wound, one peritoneal fluid and one blood) had a cefotaxime MIC of 32 µg/mL.

### 2.2. Comparison of Categorisation Results Using CLSI and EUCAST

Application of the EUCAST 2020 increased the number of resistant isolates when compared to CLSI 2015 for ceftazidime (8.6% vs. 6.0%), aztreonam (1.7% vs. 0.9%), ciprofloxacin (3.4% vs. 0%) and levofloxacin (0.9% vs. 0%) (Table 3).

### 2.3. Screening for Carbapenem-Resistant Genes

Of seven genes investigated, six genes (*bla*_KPC_, *bla*_VIM_, *bla*_NDM_, *bla*_GES-24_, *bla*_IMP-19_ and *bla*_OXA-48_) were not detected in any of the tested isolates, except for *bla*_cphA_. The presence of *bla*_cphA_ was detected in carbapenem resistant isolates with clinical origins but not observed in non-clinical isolates. Overall, more than 65% of carbapenem resistant clinical isolates carried *bla*_cphA_ in resistance phenotypes: doripenem (67.2%, 45/67), imipenem (65.9%, 54/82) and meropenem (65.2%, 30/46).

## 3. Discussion

*A. dhakensis*, increasingly recognised as an emerging human pathogen, was identified as the predominant *Aeromonas* species among clinical isolates in Malaysia (50%, 47/94) and Singapore (40.5%, 47/116). The similarity of dominant species in the two countries may be due to comparable environmental factors, such as climate, temperature and humidity, as well as dietary patterns. In this study, although the 94 clinical isolates recovered from patients in Malaysia and Singapore were previously characterised through molecular fingerprinting as not clonally related, *A. dhakensis* isolates from both countries were found to harbour a higher number of virulence genes when compared to other *Aeromonas* species [12]. All clinical isolates were positive for at least 6 of the 15 virulence genes, and these genes (*ela*, *lip*, *alt*, *ser*, *exu*, *fla* and *aer*) were present in ≥90% of the isolates (Supplementary Figure S1). The dominance of *A. dhakensis* infections in Malaysia and Singapore was postulated to be an indication of the virulence potential of this species, in accordance with findings of other studies [13,14]. Furthermore, *A. dhakensis* in both countries were revealed to be not unequivocally considered diarrhoeal pathogens, as they were frequently isolated from extraintestinal sites, i.e., most frequently from pus/wound culture in Malaysia clinical isolates and from blood culture from Singapore clinical isolates [12]. Whereas antimicrobials are usually not prescribed for self-limiting diarrhoeal infections, empirical clinical antimicrobial therapy may be considered for severe diarrhoea (e.g., bloody diarrhoea) or invasive infections (e.g., septicaemia). Recognising the clinical relevance of *A. dhakensis* and its ability to cause invasive disease, the antimicrobial resistance profiles of the *A. dhakensis* from clinical and non-clinical sources generated from this study could facilitate a better understanding of the antimicrobial resistance traits of the pathogen to support the selection of optimal treatment regimens for disease management.

Carbapenems are recognised as a critically important antimicrobial (CIA) by the World Health Organization for human medicine. Resistance to such antimicrobials is a public health concern, as it would render treatment of infection less effective and increase healthcare costs. The mortality rate of patients infected with carbapenem resistant *Aeromonas* spp. due to meropenem treatment failure was reported as 33.3% (7/21) and 100% in patients with bacteraemia [15]. Further genotypic screening revealed that more than 65% of carbapenem resistant clinical isolates carried the chromosomally encoded metallo-beta-lactamase *bla*_cphA_, suggesting an intrinsic AMR mechanism could underlie the local epidemiology of resistant *Aeromonas* infections in the investigated countries. However, the clinical isolates included in our study were from selected public hospitals and might not represent complete case data to draw a concrete conclusion on resistance patterns from both countries. In the future, more samples from more hospitals in these countries could be screened to further substantiate our current findings.

Antimicrobial resistance of clinical *A. dhakensis* to imipenem and meropenem has been reported previously in two studies in Taiwan but with lower frequencies compared to this study [4,13]. Chen et al. reported that the resistance rate of imipenem was 10.8% (4/37) using E-test strips [4]. Wu et al. documented a resistance rate of 4.2% (2/48) for both imipenem and meropenem using the broth microdilution method of the Trek Sensititre system [13]. The relatively higher carbapenem resistance rate in the clinical isolates in our study has three possible explanations:(i) different platforms with different inoculum preparations; (ii) geographically related variations in antimicrobial resistance patterns due to differences in the healthcare system, particularly in terms of treatment regimen recommended, practices of healthcare professionals and patients’ behaviour towards the use of antimicrobials; or (iii) indirect factors, such as lifestyle, seasonal difference, dietary preference, e.g., the Southeast Asia region exhibits a remarkably high per capita fish consumption of 33.4 kg compared to Asia as a whole (21.3 kg) [16]. This calls for constant monitoring of *A. dhakensis* from aquatic-related sources for public health mitigation. It also underscores that clinical empirical carbapenem therapy for *A. dhakensis* infection should be used with caution.

Among non-clinical isolates, those recovered from the gills of healthy food fish Marble Goby exhibited a relatively higher resistance rate to carbapenems than that of others (ornamental fish tank water and recreational lake water): imipenem, 100% vs. 16.7% and 100%; doripenem, 75% vs. 16.7% and 0%; and meropenem, 25% vs. 11.1% and 0% (Data not shown). The occurrence of *A. dhakensis* (12.7%, 8/63) was also reported in freshly and moribund Nile tilapia samples in India [17]. The study reported that one *A. dhakensis* isolate randomly selected for AMR profiling showed resistance towards imipenem and meropenem with an MIC >32 µg/mL via MIC strips (HiMedia), whereas, in our study, we determined the MIC of the carbapenems (imipenem and meropenem) to be up to 8 µg/mL. The detection of carbapenem resistant *A. dhakensis* in food fish revealed a potential risk to public health, as well as a potential pathway for humans to acquire resistant bacteria through consumption of aquaculture food contaminated with resistant bacteria. However, only a limited number of isolates (*n* = 4) from a relatively small sample size of food fish (*n* = 15) collected at intervals from November 2015 to October 2017 were used in the study. Further longitudinal monitoring for AMR *A. dhakensis* with larger sample size and increased sampling coverage, preferably complemented by whole-genome sequencing, should be carried out to identify possible transmission routes of *A. dhakensis.* The exceptional rate of aquaculture growth in Asia as a region should also be considered in terms of meeting the majority of the global seafood demand. Intensification of the aquaculture system might be expected to drive the usage of antimicrobials to maintain animal health. Hence, continued monitoring of resistant aeromonads is warranted using a larger sample size in the environmental niches that the bacterium possibly inhabits.

Among the three carbapenems, the AST for meropenem of the 94 clinical isolates and 4 non-clinical isolates from food fish (Marble Goby) was previously reported using the Kirby–Bauer disk diffusion method [11,12]. However, in these studies, only one meropenem resistant clinical isolate was detected, and no resistance was observed in food fish isolates. Possible explanations for the discrepancy between the results of microdilution and disk diffusion assays could be the concentrations of antimicrobial agents and different inoculum preparation methods. MicroScan Prompt (the microdilution method used in this study) preparation offers a standardised inoculum (generally expected inoculum size: 6.9 × 10^5^ cfu/mL), whereas the disk diffusion test requires manual preparation with inoculums adjusted to match a 0.5 MacFarland turbidity standard (10^4^–10^5^ cfu/mL). The determination of in vitro susceptibility of carbapenemase-producing aeromonads by disk diffusion or dilution techniques usually yields a susceptible genotype unless using a large inoculum (3 × 10^8^ cfu/mL) [18]. For the detection of meropenem resistance in *A. dhakensis*, Sinclair et al. reported a low accuracy of disk diffusion (2.6%, 1/39) and E-test (0%) compared to the microdilution method (61.5%, 24/39) [19]. By using the microdilution method via the MicroScan system, the emergence of carbapenem resistance of *Aeromonas* spp., including *A. dhakensis*, was reported in Colombia [15]. This observation underscores the importance of selecting an appropriate method for the examination of carbapenem resistance in *Aeromonas*.

Each AST detection platform has inherent strengths and limitations. As shown in this study, most MIC results from MicroScan for nine antimicrobial agents—piperacillin/tazobactam, cefuroxime, cefoxitin, ertapenem, gentamicin, tetracycline, ciprofloxacin, levofloxacin and chloramphenicol—were outside the reference MIC range of CLSI. These MIC results were categorised as “not determinable”, as the MICs could not be assessed using CLSI 2015 breakpoints (Table 3). A high rate of “not determinable” was observed in cefoxitin (82.9%) and ertapenem (62.4%) (Table 1). This also calls for continuous improvement of commercial platforms to incorporate the latest antimicrobial breakpoints [20].

The kappa statistics for *A. dhakensis* showed almost perfect agreement (kappa value: 0.810–1.000) between CLSI 2015 and EUCAST 2020 for cefepime, ceftazidime and trimethoprim/sulfamethoxazole. There is no interpretative breakpoint for carbapenem resistance provided by EUCAST 2020; thus, the agreement of carbapenem resistance between EUCAST and CLSI methods was not discussed in this study. For ciprofloxacin and levofloxacin, kappa analysis revealed no agreement (kappa value = 0), as four clinical isolates with MIC >2 µg/mL interpreted as susceptible to ciprofloxacin by CLSI 2015 were categorised as resistant by EUCAST 2020. On the other hand, a clinical isolate with >4 µg/mL interpreted as susceptible to levofloxacin by CLSI 2015 was categorised as resistance by EUCAST 2020. Overall, EUCAST 2020 has a more stringent breakpoint for susceptibility for selected antimicrobial agents compared to CLSI 2015 (Table 3). The stringent breakpoint might help to curb the inappropriate use of antibiotics and control the rising rate of AMR, but this may require further harmonisation, as this has implications for laboratories considering switching between CLSI and EUCAST, as well as for a large-scale AMR surveillance comparing data within and between countries.

Further investigation of the genetic background of carbapenem resistant isolates in our study revealed a chromosomally encoded metallo-β-lactamase *cphA* gene, indicating that these clinical isolates are attributed to intrinsic resistance. This finding is in agreement with previously published reports on *A. dhakensis* isolated from human infections in Australia [19], Taiwan [20] and China [21]. The results suggest that clinical use of carbapenem monotherapy should be considered with caution in order to avoid potential treatment failure and that alternative antimicrobial treatment options, such as fourth-generation cephalosporins, quinolones, amikacin, aztreonam and trimethoprim/sulfamethoxazole, remain active for most *A. dhakensis* isolates.

## 4. Materials and Methods

### 4.1. Bacterial Isolates

A total of 117 *A. dhakensis* isolates were retrieved from previous studies [7,8,9,10,11,12,22]. These isolates consisted of 94 clinical isolates from Malaysia and Singapore and 23 non-clinical isolates from Malaysia (Table 4). Briefly, these isolates were recovered using *Aeromonas* selective agar, and their genus identities were confirmed using *GCAT* gene and species level using *rpoD* gene sequencing. For non-clinical strains, non-replicate isolate clones were confirmed using enterobacterial repetitive intergenic consensus PCR. The bacterial isolates were revived from glycerol stock onto 3 mL of LB broth and incubated at 35 °C for 20 h with agitation. The next day, bacterial suspensions were subcultured on LB agar and incubated at 35 °C for 20 h.

### 4.2. Antimicrobial Susceptibility Testing

The isolates were subjected to antimicrobial susceptibility testing by the broth microdilution using a MicroScan plate (Beckman Coulter, CA, USA). Four well-isolated colonies were selected using an inoculation wand of the Prompt Inoculation System D and inoculated into a diluent. Following mixing, 115 µL of bacterial suspension was added to each well of the MicroScan plate. The plates were incubated at 35 °C for 20 h, and MIC values were determined manually, followed by susceptibility categorisation based on the CLSI and EUCAST guidelines [23,24]. *Escherichia coli* ATCC 25922 and *Pseudomonas aeruginosa* ATCC 27853 were used as quality-control organisms as per the manufacturer’s protocol.

### 4.3. Screening for Carbapenem-Resistant Genes

The genomic DNA of carbapenem resistant *A. dhakensis* isolates was extracted using the boiling method [25]. These isolates were screened for seven carbapenem-related genes, including *bla*_cphA_, *bla*_KPC_, *bla*_VIM_, *bla*_NDM_, *bla*_GES-24_, *bla*_IMP-19_ and *bla*_OXA-48_, using primers and conditions as described in published studies (Supplementary Table S1). A representative of a positive isolate was randomly chosen and confirmed by direct DNA sequencing.

## 5. Conclusions

In summary, our study revealed a high prevalence of carbapenem resistance in *A*. *dhakensis* clinical isolates, and most of these isolates were found to harbour the *bla*_cphA_ gene, which is linked to intrinsic resistance. Given their inherent nature, *A. dhakensis* infections could be difficult to be treated with carbapenem monotherapy before screening. Therefore, the reliable identification of carbapenemase-producing isolates is an important first step in prescribing an appropriate drug in order to prevent the development of untreatable infections.

## Figures and Tables

**Table 1:** Antibiotic susceptibility profile of clinical and non-clinical *Aeromonas dhakensis*.

Antimicrobial Class	Antimicrobial Agent	Interpretative Break Points ^#^ (µg/mL)	Total (*n* = 117)	Clinical Isolates (*n* = 94)			Non-Clinical Isolates (*n* = 23)			*p* Value	ND
		Susceptible/Resistant	R *n* (%)	S *n* (%)	I *n* (%)	R *n* (%)	S *n* (%)	I *n* (%)	R *n* (%)		
**Penicillins and beta-lactam**	Piperacillin/Tazobactam	≤16/4/≥128/4	0 (0)	83 (88.3)	1 (1.1)	0 (0)	16 (69.6)	5 (21.7)	0 (0)	1	12 (10.3)
**Cephems**	Cefuroxime	≤8/≥32	0 (0)	83 (88.3)	2 (2.1)	0 (0)	21 (91.3)	2 (8.7)	0 (0)	1	9 (7.7)
	Cefotaxime	≤1/≥4	10 (8.5)	81 (86.2)	4 (4.3)	9 (9.6)	19 (82.6)	3 (13.0)	1 (4.3)	0.6847	0 (0)
	Cefoxitin	≤8/≥32	0 (0)	9 (9.6)	7 (7.4)	0 (0)	1 (4.3)	2 (8.7)	0 (0)	1	97 (82.9)
	Ceftazidime	≤4/≥16	7 (6.0)	85 (90.4)	2 (2.1)	7 (7.4)	22 (95.7)	1 (4.3)	0 (0)	0.3428	0 (0)
	Cefepime	≤4/≥16	2 (1.7)	90 (95.7)	2 (2.1)	2 (2.1)	23 (100)	0 (0)	0 (0)	1	0 (0)
**Carbapenems**	Doripenem	≤1/≥4	73 (62.4)	17 (18.1)	10 (10.6)	67 (71.3)	15 (65.2)	2 (8.7)	6 (26.1)	0.0001 *	0 (0)
	Ertapenem	≤0.5/≥2	0 (0)	13 (13.8)	14 (14.9)	0 (0)	14 (60.9)	2 (8.7)	0 (0)	1	73 (62.4)
	Imipenem	≤1/≥4	90 (76.9)	11 (11.7)	1 (1.1)	82 (87.2)	15 (65.2)	0 (0)	8 (34.8)	0.0001 *	0 (0)
	Meropenem	≤1/≥4	49 (41.9)	29 (30.9)	19 (20.2)	46 (48.9)	18 (78.3)	2 (8.7)	3 (13.0)	0.0019 *	0 (0)
**Monobactams**	Aztreonam	≤4/≥16	1 (0.9)	93 (98.9)	1 (1.1)	0 (0)	22 (95.7)	0 (0)	1 (4.3)	0.1949	0 (0)
**Aminoglycosides**	Amikacin	≤16/≥64	0 (0)	93 (98.9)	1 (1.1)	0 (0)	21 (91.3)	2 (8.7)	0 (0)	1	0 (0)
	Gentamicin	≤4/≥16	0 (0)	85 (90.4)	1 (1.1)	0 (0)	22 (95.7)	1 (4.3)	0 (0)	1	8 (6.8)
**Tetracyclines**	Tetracycline	≤4/≥16	0 (0)	60 (63.8)	5 (5.3)	0 (0)	20 (87.0)	0 (0)	0 (0)	1	32 (27.4)
**Fluoroquinolones**	Ciprofloxacin	≤1/≥4	0 (0)	91 (96.8)	0 (0)	0 (0)	22 (95.7)	0 (0)	0 (0)	1	4 (3.4)
	Levofloxacin	≤1/≥8	0 (0)	93 (98.9)	0 (0)	0 (0)	23 (100)	0 (0)	0 (0)	1	1 (0.9)
**Folate Pathway Inhibitors**	Trimethoprim/Sulfamethoxazole	≤2/38/≥4/76	13 (11.1)	82 (87.2)	0 (0)	12 (12.8)	22 (95.7)	0 (0)	1 (4.3)	0.4588	0 (0)
**Phenicols**	Chloramphenicol	≤8/≥32	0 (0)	85 (90.4)	4 (4.2)	0 (0)	21 (91.3)	2 (8.7)	0 (0)	1	5 (4.3)

Interpretative break points: ^#^—based on CLSI guideline M45-A3 (2015); ND—not determinable, as the MicroScan MIC ranges are outside the CLSI breakpoints (2015); S—susceptible; I—intermediate; R—resistant; *—statistical significance at the 5% level (fisher exact test with a 2 × 2 contingency table).

**Table 2:** Comparison of antibiotic susceptibility profiles of clinical *Aeromonas dhakensis* isolates between Malaysia and Singapore.

Antimicrobial Agent		Malaysia (47 Isolates) *n* (%)	Singapore (47 Isolates) *n* (%)	Total (94 Isolates) *n* (%)
Cefotaxime				
	Susceptible	46 (97.9)	35 (74.5)	81 (86.2)
	Intermediate	1 (2.1)	3 (6.4)	4 (4.3)
	Resistance	0	9 (19.1)	9 (9.6)
Ceftazidime				
	Susceptible	47 (100)	38 (80.9)	85 (90.4)
	Intermediate	0	2 (4.3)	2 (2.1)
	Resistance	0	7 (14.9)	7 (7.4)
Cefepime				
	Susceptible	47 (100)	43 (91.5)	90 (95.7)
	Intermediate	0	2 (4.3)	2 (2.1)
	Resistance	0	2 (4.3)	2 (2.1)
Doripenem				
	Susceptible	9 (19.1)	8 (17.0)	17 (18.1)
	Intermediate	5 (10.6)	5 (10.6)	10 (10.6)
	Resistance	33 (70.2)	34 (72.3)	67 (71.3)
Imipenem				
	Susceptible	6 (12.8)	5 (10.6)	11 (11.7)
	Intermediate	0	1 (2.1)	1 (1.1)
	Resistance	41 (87.2)	41 (87.2)	82 (87.2)
Meropenem				
	Susceptible	15 (31.9)	14 (29.8)	29 (30.9)
	Intermediate	9 (19.1)	10 (21.3)	19 (20.2)
	Resistance	23 (48.9)	23 (48.9)	46 (48.9)
Aztreonam				
	Susceptible	47 (100)	46 (97.9)	93 (98.9)
	Intermediate	0	1 (2.1)	1 (1.1)
	Resistance	0	0	0
Trimethoprim/Sulfamethoxazole				
	Susceptible	40 (85.1)	42 (89.4)	82 (87.2)
	Intermediate	0	0	0
	Resistance	7 (14.9)	5 (10.6)	12 (12.8)

**Table 3:** Differences between *Aeromonas dhakensis* isolate susceptibilities to various antimicrobials in accordance with CLSI 2015 and EUCAST 2020 recommendations.

Antimicrobial Agent	Susceptible/Resistant MIC Points		Susceptible			Intermediate			Resistant		
	CLSI 2015	EUCAST 2020	CLSI 2015	EUCAST 2020	Kappa Value	CLSI 2015	EUCAST 2020	Kappa Value	CLSI 2015	EUCAST 2020	Kappa Value
Cefepime	≤4/≥ 16	≤1/>4	113	112	0.885	2	0	0	2	2	1.000
Ceftazidime	≤4/≥16	≤1/>4	107	106	0.948	3	0	0	7	10	0.810
Aztreonam	≤4/≥ 16	≤1/>4	115	114	0.796	1	0	0	1	2	0.663
Ciprofloxacin	≤1/≥ 4	≤0.25/>0.5	113	0	0	0	0	0	0	4	0
Levofloxacin	≤1/≥ 8	≤0.25/>1	116	0	0	0	0	0	0	1	0
Trimethoprim/sulfamethazole	≤2/38/≥ 4/76	≤2/ 4	104	104	1.000	0	0	0	13	10	0.856

**Table 4:** *Aeromonas dhakensis* isolates used in this study.

Isolates	Origins	Malaysia	Singapore
**Clinical**	Stool	27	13
	Peritoneal fluid	5	1
	Pus/wound	13	8
	Urine	1	1
	Others *	1	4
	Blood		10
	Bile		6
	Tissue		3
	Sputum		1
	**Total**	**47**	**47**
**Non-clinical**	Ornamental fish tank water	18	
	Freshwater recreational lake	1	
	Food fish (Marble Goby)	4	
	**Total**	**23**	

* Others from lung, fluid and stent.

## Supplementary Materials

The following supporting information can be downloaded at: https://www.mdpi.com/article/10.3390/pathogens11080833/s1, Figure S1: Primers sequences used for carbapenem resistant genes detection; Table S1: Heatmap of presence/absence of the 15 genes with variable pattern of virulence among clinical Aeromonas dhakensis. References [26,27,28,29,30] are mentioned in Supplementary file.
