# Supplementary material for: Aeromonas dhakensis: Clinical Isolates with High Carbapenem Resistance
Source: Pathogens. 2022 Jul 26;11(8):833. doi: 10.3390/pathogens11080833 (PMC9394330; doi:10.3390/pathogens11080833)
Supplement: Supplementary file 1 [file pathogens-11-00833-s001.zip › pathogens-1765496-supplementary.pdf]

Supplementary Table S1. Primers sequences used for carbapenem resistant genes detection.

Gene	Sequence (5′ - 3′)	Amplicon size (bp)	Reference
*bla*cphA	F: GCTTAGAGCTCCTAAGGAGCAAGATGAAAGGTTGG<br>R: GCATAGGTACCTTATGACTGGGGTGCGGCCTTG	720	Wu et al., 2012
*bla*KPC	F: GCAACAACGACAATCCATCCT<br>R: GGGATAGGCGTAACTCTCCCAA	538	Dallenne et al., 2010
*bla*VIM	F: GATGGTGTTTGGTCGCATA<br>R: CGAATGCGCAGCACCAG	390	Dallenne et al., 2010
*bla*NDM	F: GGTTTGGCGATCTGGTTTTC<br>R: CGGAATGGCTCATCACGATC	621	Poirel et al., 2011
*bla*GES-24	F: GCTTCATTCACGCACTATT<br>R: CGATGCTAGAAACCGCTC	323	Poirel et al., 2000
*bla*IMP-19	F: GTTTTATGTGTATGCTTCC<br>R: AGCCTGTTCCCATGTAC	678	Neuwirth et al., 2007
*bla*OXA-48	F: TTGGTGGCATCGATTATCGG<br>R: GAGCACTTCTTTTGTGATGGC	744	Szekely et al., 2013

- Dallenne. C,; Da Costa. A.; Decré. D.; Favier. C.; Arlet. G. Development of a set of multiplex PCR assays for the detection of genes encoding important beta-lactamases in Enterobacteriaceae. J. Antimicrob. Chemother. **2010**,65:490-495.
- Poirel. L.; Walsh. T.R.; Cuvillier. V.; Nordmann. P. Multiplex PCR for detection of acquired carbapenemase genes. Diagn. Microbiol. Infect. Dis. **2011,**70:119-123.
- Poirel, L.; Le Thomas. I.; Naas. T.; Karim. A.; Nordmann. P. Biochemical sequence analyses of GES-1, a novel class A extended-spectrum beta-lactamase, and the class 1 integron In52 from *Klebsiella pneumoniae.* Antimicrob. Agents Chemother. **2000**,44;622-632.
- Neuwirth. C.; Siebor. E.; Robin. F.; Bonnet. R. First occurrence of an IMP metallo-beta-lactamase in *Aeromonas caviae*: IMP-19 in an isolate from France. Antimicrob. Agents Chemother. **2007**.51:4486-4488.
- Szekely. E.; Damjanova. I.; Janvari. L.; Vas. K.E.; Molnar. S.; Bilca. D.V.; Lorinczi. L.K.; Toth. A. First description of bla(NDM-1), bla(OXA-48), bla(OXA-181) producing Enterobacteriaceae strains in Romania. Int. J. Med. Microbiol. **2013**.303:697-700.
- Wu. C.J.; Chen. P.L.; Wu. J.J.; Yan. J.J.; Lee. C.C.; Lee. H.C.; Lee. N.Y.; Chang. C.M.; Lin. Y.T.; Chiu. Y.C.; Ko. W.C. Distribution and phenotypic and genotypic detection of a metallo-β-lactamase, *CphA*, among bacteraemic *Aeromonas* isolates. J. Med. Microbiol. **2012**,61(Pt5):712-719.

Supplementary Figure S1. Heatmap of presence/absence of the 15 genes with variable pattern of virulence among clinical *Aeromonas dhakensis.*

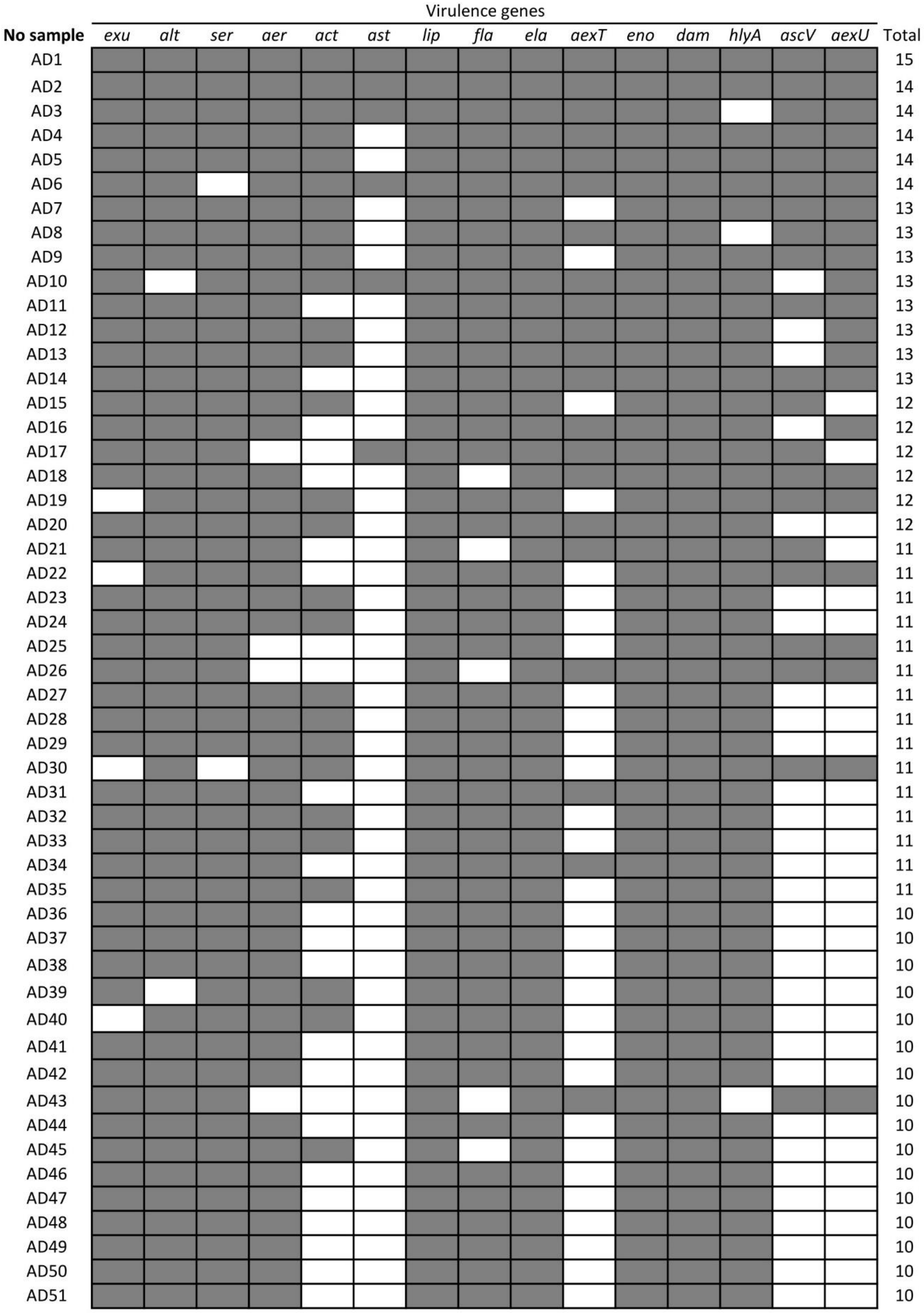

	Virulence genes															
**No sample**	*exu*	*alt*	*ser*	*aer*	*act*	*ast*	*lip*	*fla*	*ela*	*aexT*	*eno*	*dam*	*hlyA*	*ascV*	*aexU*	Total
AD52	■	■	■	■			■	■	■		■	■	■			10
AD53	■	■	■	■			■	■	■		■	■	■			10
AD54	■	■	■	■			■	■	■		■	■	■			10
AD55	■	■	■	■			■	■	■		■	■	■			10
AD56		■	■	■			■	■	■		■	■	■			9
AD57	■	■		■			■	■	■		■	■	■			9
AD58	■	■	■				■	■	■		■	■	■			9
AD59	■	■	■	■		■	■	■	■	■						9
AD60	■	■	■	■			■	■	■		■		■			9
AD61		■	■	■			■	■	■		■	■	■			9
AD62	■	■	■	■			■	■	■			■	■			9
AD63	■	■	■	■			■	■	■	■		■				9
AD64	■	■	■				■	■	■		■	■	■			9
AD65	■	■	■	■		■	■	■	■	■						9
AD66	■	■		■			■	■	■		■	■	■			9
AD67	■			■	■		■	■	■		■		■			9
AD68	■	■		■			■		■		■	■	■			8
AD69	■	■	■	■			■	■	■	■						8
AD70	■	■	■	■				■	■		■	■				8
AD71	■	■	■	■	■		■	■	■							8
AD72	■	■	■	■			■	■	■	■						8
AD73	■	■	■	■			■	■	■	■						8
AD74	■	■	■	■	■		■	■	■							8
AD75	■	■	■	■	■		■	■	■							8
AD76	■	■	■	■	■		■	■	■							8
AD77	■			■			■	■	■	■		■				8
AD78	■	■	■				■	■	■		■					7
AD79	■	■	■				■	■	■			■				7
AD80	■	■	■	■			■	■	■							7
AD81	■	■	■	■			■	■	■							7
AD82	■	■	■	■			■	■	■							7
AD83	■	■	■	■			■	■	■							7
AD84	■	■	■	■			■	■	■							7
AD85	■	■	■	■			■	■	■							7
AD86	■	■	■	■			■	■	■							7
AD87	■	■	■	■			■	■	■							7
AD88	■	■	■	■			■	■	■							7
AD89	■	■	■				■	■	■	■						7
AD90	■	■	■	■		■		■	■							7
AD91	■	■	■	■			■	■	■							7
AD92	■	■	■	■			■	■	■							7
AD93	■	■	■	■			■	■	■							7
AD94	■	■	■	■			■	■								6
Positive (%)	88 (94)	90 (96)	89 (95)	85 (90)	32 (34)	8 (9)	92 (98)	88 (94)	93 (99)	29 (31)	66 (70)	67 (71)	62 (66)	21 (22)	22 (23)	
